# Hallmarks of the cancer cell of origin: Comparisons with “energetic” cancer stem cells (e-CSCs)

**DOI:** 10.18632/aging.101822

**Published:** 2019-02-13

**Authors:** Federica Sotgia, Marco Fiorillo, Michael P. Lisanti

**Affiliations:** 1 Translational Medicine, School of Environment and Life Sciences, Biomedical Research Centre (BRC), University of Salford, Greater Manchester M5 4WT, United Kingdom; 2 The Department of Pharmacy, Health and Nutritional Sciences, The University of Calabria, Cosenza, Italy

**Keywords:** cancer cell of origin, cancer stem cells (CSCs), senescence, metabolism, anti-oxidant response, tamoxifen resistance

## Abstract

Here, we discuss the expected hallmark(s) of the cancer cell of origin and how this may be related to a new tumor cell phenotype, namely “energetic” cancer stem cells (e-CSCs). e-CSCs show many features that would be characteristic of the cancer cell of origin, including the over-expression of p21-WAF (CDKN1A), a key marker of senescence. It is tempting to speculate that the cancer cell of origin and e-CSCs are closely related entities. e-CSCs possess a hybrid phenotype, sharing key hallmarks of senescence, “stemness” and cancer. e-CSCs are hyper-proliferative and have elevated mitochondrial metabolism, with an NRF2-mediated anti-oxidant response signature, including glutaredoxin (GLRX) and ALDH3A1 over-expression, possibly related to their escape from senescence. Finally, in e-CSCs, BCAS1 (Breast carcinoma-amplified sequence-1) protein expression was up-regulated by >100-fold. BCAS1 is a candidate oncogene associated with “stemness” and aggressive oncogenic behavior, such as Tamoxifen resistance.

## Hallmarks: cancer cell of origin vs. e-CSCs

Cancer cells are believed to originate from senescent cells that have undergone stress-induced cell cycle arrest [1–3]. During chronological aging, genetic mutations are thought to accumulate in the stem cell compartment, resulting in i) oncogene activation, ii) tumor suppressor inactivation, as well as iii) a variety of genetic chromosomal rearrangements (deletions, translocations and duplications) and other epigenetic modifications [4–6].

**Figure 1:**
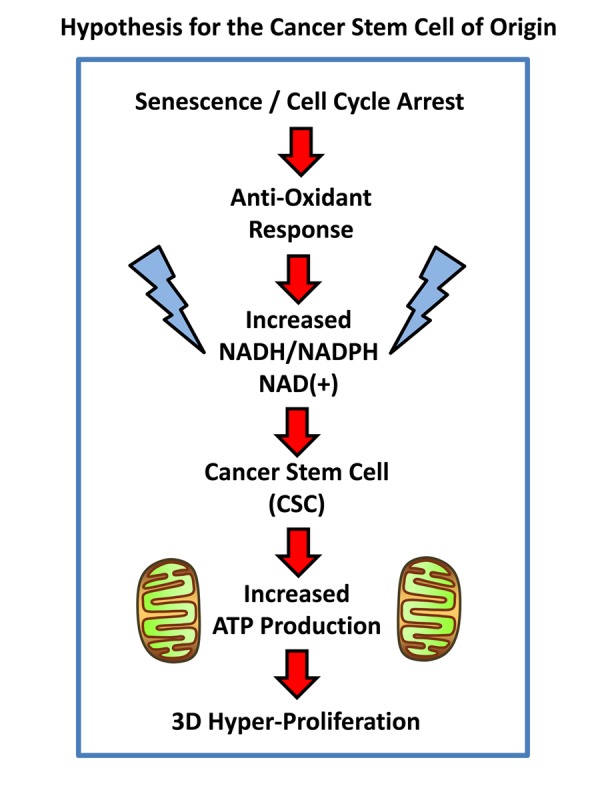
**Hypothesis for how senescent cells can mechanistically become cancer stem cells.** Senescent cells undergoing cell cycle arrest mount an anti-oxidant defense, to increase their levels of NADH. In turn, increased NADH levels are known to be sufficient to rescue senescent cells from cell cycle arrest, allowing new cell proliferation, by “re-activating” or “resuscitating” senescent cells. Increased mitochondrial power would then drive elevated ATP production and 3D anchorage-independent growth, fostering the generation and propagation of the cancer cell of origin.

Presumably, cancer cells may arise from senescent cells through a process of re-activation, leading from cell cycle arrest to hyper-proliferation. It has been proposed that telomerase activity (hTERT) catalyzes this transition [3]. However, this process of re-activation can also be achieved through cellular metabolism [7,8]. For example, David Sinclair and his colleagues at Harvard Medical School have shown that senescent cells suffer from a deficiency of NADH (nicotinamide adenine dinucleotide), a key anti-oxidant, and that senescent cells can be revived simply by the addition of an NADH precursor metabolite (namely, nicotinamide riboside) to the cell culture media [7,8]. These results directly imply that the anti-oxidant response may also trigger the revival of senescent cells (Figure 1). Similarly, cancer stem cells (CSCs) are strictly dependent on NADH, for their propagation as 3D-spheroids [9]. Also, CSCs have been shown to over-express classical “embryonic” markers of stemness, such as Oct4, c-Myc and Nanog, among others.

**Figure 2:**
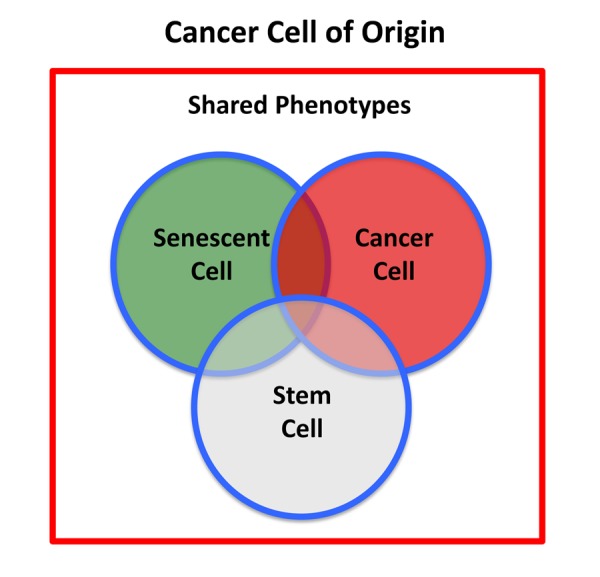
**Cancer stem cell of origin.** The cancer stem cell of origin would be predicted to have a chimeric- or hybrid-phenotype, retaining elements of i) senescent cells, ii) cancer cells, and iii) stem cells, as we observe in e-CSCs.

Therefore, the cancer stem cell of origin [10,11] would be predicted to retain certain properties of senescent stem cells, while undergoing a gain-of-function process, thereby obtaining new properties of a cancer cell, resulting in a chimeric or hybrid phenotype (Figure 2). These properties would be expected to include: biological markers of senescence; a hyper-proliferative phenotype; a very active metabolic program to support anabolic growth and proliferation; an anti-oxidant response, for driving the revival program, to overcome senescence-induced cell cycle arrest [7,8,12]; and key stem cell features (See Table 1, Left).

**Table 1:** Expected hallmarks of the cancer cell of origin: Comparison with e-CSCs.

**Cell Type: **	**Cancer Cell of Origin**	**e-CSCs**
**Properties:**	**Expected Features**	**Observed Features**
**1) Cell Cycle Arrest:**	**Senescence Markers**	**Elevated p21-WAF (~17-fold)**
**2) Propagation:**	**Hyper-Proliferative **	**G0/G1: ~35-37% S-phase: ~10-18%**
		**G2/M: ~32-33% Polyploid: ~12-17%**
**3) Metabolism:**	**Metabolically Active**	**Increased Mitochondrial Mass (~4-fold); High OXPHOS & Glycolysis**
**4) REDOX:**	**Anti-Oxidant Response**	**ALDH Functional Activity (~9-fold Increased)**
	**[Produces NADH]**	**Glutaredoxin-1 (GLRX) (~11-fold Increased)**
		**ALDH3A1 (~10-fold Increased)**
		**QPRT (~4-fold Increased)**
		**RRM2, GCLC, NQO2 (Each ~2-fold Increased)**
**5) “Stemness”:**	**Stem Cell Markers**	**High Flavin-based Auto-fluorescence (FAD/FMN); Large Cell Size; Aldefluor (+); Anchorage-Independence; **
		**BCAS1 (+) (>100-fold Increased)**
**6) Inhibitors:**	**Unknown**	**Mitochondrial OXPHOS Inhibitors and CDK4/6 Inhibitors**

Recently, our laboratory may have fortuitously isolated a new tumor cell with a cancer cell of origin phenotype, by using flavin-derived auto-fluorescence as a selection marker, via flow-cytometry [13]. To functionally describe these cells, we coined the term “energetic” cancer stem cells (e-CSCs) [13]. Briefly, e-CSCs retain high expression of the senescence marker p21-WAF (CDKN1A), while paradoxically manifesting a hyper-proliferative phenotype (Table 1, Right). Based on Ingenuity Pathway Analysis (IPA) of e-CSC proteomics data, other upstream regulators of cell cycle arrest and senescence were activated, including p53, TGFB1, and p38 MAPK signaling [13].

**Figure 3:**
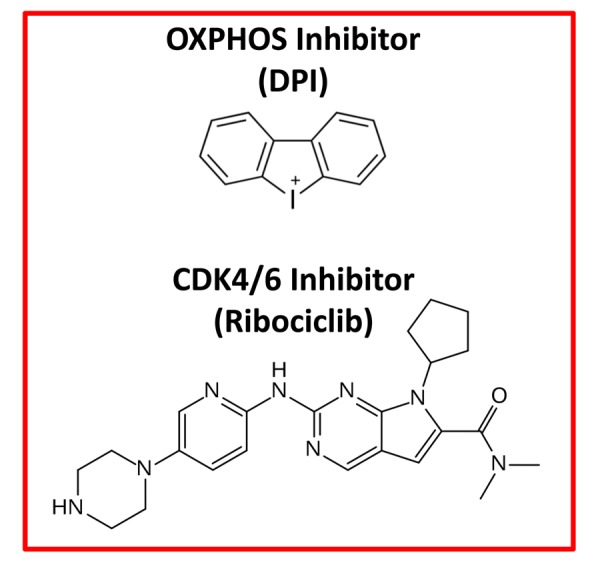
**Therapeutic targeting of e-CSCs.** Experimentally, both an OXPHOS inhibitor (Diphenyleneiodonium; DPI) and a CDK4/6 (Ribociclib) inhibitor were effective in abrogating the 3D-propagation of e-CSCs.

Moreover, e-CSCs show a hyper-metabolic phenotype, with increased mitochondrial mass, elevated oxidative mitochondrial metabolism, as well as enhanced glycolytic function. e-CSCs mount a strong anti-oxidant response, characterized by increased levels of glutaredoxin-1 (GLRX) expression and ALDH isoform activity (Table 1, Right). Interestingly, e-CSCs also show a number of stem-like features, including large cell size and anchorage-independent growth, that is highly sensitive to inhibitors of both mitochondrial OXPHOS and CDK4/6 cell cycle progression (Figure 3). In addition, large cell size is a key feature commonly shared by CSCs, e-CSCs and senescent cells alike [13–16].

Lastly, in e-CSCs, BCAS1 (Breast carcinoma-amplified sequence-1) protein expression was up-regulated, by nearly 120-fold [13]. BCAS1 over-expression [17] is functionally associated with “stemness” [18], a more aggressive cancer cell phenotype [19] and Tamoxifen-resistance [20,21]. MCF7-TAMR cells, which were chronically selected for Tamoxifen-resistance, by including Tamoxifen in their tissue culture media, show a >50-fold increase in BCAS1 protein expression, promoting increased ATP production [21].

## Conclusions

In summary, e-CSCs possess many of the predicted hallmarks that would be expected of the cancer cell of origin. This assertion could have broad implications for mechanistically understanding and more effectively targeting the cancer stem cell of origin, to prevent tumor recurrence and metastatic dissemination, throughout the body, to significantly improve clinical outcomes in cancer therapy.
